# Influence of Dopamine on Fluorescent Advanced Glycation End Products Formation Using *Drosophila melanogaster*

**DOI:** 10.3390/biom11030453

**Published:** 2021-03-17

**Authors:** Ana Filošević Vujnović, Katarina Jović, Emanuel Pištan, Rozi Andretić Waldowski

**Affiliations:** 1 Department of Biotechnology, University of Rijeka, 51000 Rijeka, Croatia; ana.filosevic@biotech.uniri.hr; 2 Faculty of Health and Medical Sciences, University of Surrey, Guildford, Surrey GU2 7XH, UK; kj00230@surrey.ac.uk; 3 High School Andrija Mohorovičić, 51000 Rijeka, Croatia; emanuelpistan@gmail.com

**Keywords:** dopamine (DA), fluorescent advanced glycation end products (fAGEs), psychostimulants, oxidation, *Drosophila melanogaster*

## Abstract

Non-enzymatic glycation and covalent modification of proteins leads to Advanced Glycation End products (AGEs). AGEs are biomarkers of aging and neurodegenerative disease, and can be induced by impaired neuronal signaling. The objective of this study was to investigate if manipulation of dopamine (DA) in vitro using the model protein, bovine serum albumin (BSA), and in vivo using the model organism *Drosophila melanogaster,* influences fluorescent AGEs (fAGEs) formation as an indicator of dopamine-induced oxidation events. DA inhibited fAGEs-BSA synthesis in vitro, suggesting an anti-oxidative effect, which was not observed when flies were fed DA. Feeding flies cocaine and methamphetamine led to increased fAGEs formation. Mutants lacking the dopaminergic transporter or the D1-type showed further elevation of fAGEs accumulation, indicating that the long-term perturbation in DA function leads to higher production of fAGEs. To confirm that DA has oxidative properties in vivo, we fed flies antioxidant quercetin (QUE) together with methamphetamine. QUE significantly decreased methamphetamine-induced fAGEs formation suggesting that the perturbation of DA function in vivo leads to increased oxidation. These findings present arguments for the use of fAGEs as a biomarker of DA-associated neurodegenerative changes and for assessment of antioxidant interventions such as QUE treatment.

## 1. Introduction

Advanced Glycation End products (AGEs) are heterogeneous groups of compounds associated with diverse pathophysiological conditions, from aging and neurodegeneration to the impairment of endogenous antioxidant defense [1,2,3,4,5]. It is still not known if endogenous AGEs cause, or are the consequence of, observed complications associated with different diseases. Correlation between fluorescent AGEs (fAGEs) accumulation and pathophysiology of several neuropsychiatric diseases was shown [6,7], but a biological mechanism between monoamine perturbation and fAGEs formation in neurodegenerative diseases is lacking.

AGEs are final products of complex multistep glycation reactions that involve irreversible, non-enzymatic covalent bonding of reductive sugars to side chains of amino acids in proteins [8,9]. A free amino group defines the site of glycation within the protein structure, while the reaction is non-specific and non-selective with respect to the reductive sugar. Based on their chemical properties, AGEs can be classified in three groups: fluorescent cross-linking AGEs based on aromatic chemical structures, non-fluorescent cross-linking AGEs and non-fluorescent non-crosslinking AGEs [10]. AGEs can be intracellular and extracellular, and are mainly associated with long-lived proteins [3,11]. Due to their chemical diversity, the non-specificity and non-selectivity of the glycation process, and aggregation potential, it is challenging to quantify total AGEs abundance in biological samples [12], while auto-fluorescence properties of fAGEs enable fast detection and quantifications [7,13].

Endogenous AGEs abundancy can be influenced by the diet, in particular, the amount of reducing sugars in the diet [14,15]. Diet rich in antioxidants leads to decreased AGEs formation both in vitro and in vivo, implicating redox regulation and blood glucose as potential modulatory factors [16,17]. Quercetin (QUE), a plant flavonoid, has antioxidant properties and leads to the inhibition of AGEs formation [17], and has shown a beneficial effect in diabetes, neurodegenerative diseases and addiction [18].

By some accounts, fAGEs accumulation is a consequence of the impairment in the dopaminergic (DA) signaling [19,20,21,22,23]. DA is a neuronal and hormonal regulator, and disruption in DA transport and signaling is the base for many neuropathological conditions raging from neurodegeneration to addiction [24,25,26]. As DA is one of the major neurotransmitters that undergoes long-term modulation after administration of addictive substances, in our experiments, we used psychostimulants cocaine (COC) and methamphetamine (METH). DA regulates neuronal plasticity after the administration of addictive substances leading to behavioral changes that characterize addictive behavior [27]. However, fAGEs formation in the context of addictive substances administration that cause protein modification with non-functional or neurotoxic consequence has not been extensively studied [28].

Genomes of *Drosophila melanogaster* and mammals share a significant degree of homology, leading to the successful use of *Drosophila* in translational medicine. The dopaminergic system in *Drosophila* shares many parallels with the mammalian system, and both in flies and mammals, dopaminergic cells represent only a small fraction of neurons in the whole brain. In flies, approximately 100,000 neurons constitute protocerebrum, out of which around 130 are dopaminergic [29]. They are organized in 8 major clusters which project broadly and have widespread effects [30,31]. Dopaminergic cells and their projections have been described in the context of a wide array of behaviors that affect: arousal, sleep, locomotor activity, addiction, learning and memory, attention, decision making, appetite and more [32]. The specificity of action of dopaminergic cells with the general effect on brain functioning is best illustrated by the finding that a single neuron from each of the PPL1 clusters is responsible for controlling wakefulness and sleep [33].

Considering that many psychiatric diseases correlate with dysfunctional dopaminergic systems and show increased AGEs formation we tested the hypothesis that genetic or pharmacological manipulations of DA will lead to change in fAGEs formation. We hypothesized that the amount of fAGEs will be modulated as a consequence of pro- or anti-oxidant effects of DA. We focused our work on fAGEs, as they are easy to quantify from the tissue sample using fluorescence emission [34]. Using bovine serum albumin (BSA) as a model protein, and glucose (GLU) as a reductive sugar, we adapted in vitro protocol for fAGEs-BSA synthesis and quantification [34,35]. We tested how the addition of DA influences the amount of fAGEs-BSA, then used pharmacological and genetic approaches to change the DA amount or signaling to correlate the effect on fAGEs formation. We show that in *Drosophila*, fAGEs formation changes under the influence of the dopaminergic signaling, and that this correlation is likely linked to the disrupted oxidative balance.

## 2. Materials and Methods

### 2.1. In Vitro Standard Calibrator fAGEs-BSA Synthesis and Characterization

#### 2.1.1. Chemicals and Reagents

3,4-dihydroxy-L-phenylalanine (L-DOPA) (≥98%), dopamine hydrochloride (DA) (≥98%), octopamine hydrochloride (OCT) (≥95%), L-tryptophan (TRY) (≥99,5%), tyramine hydrochloride (TYRA) (≥98%), quercetin dihydrate (QUE) (≥95%), the psychostimulants cocaine hydrochloride (COC) (≥97.5%) and methamphetamine hydrochloride (METH) (≥98%), bovine serum albumin (BSA) (≥98%) lyophilized and crystallized, were all purchased from Sigma Aldrich. Disodium hydrogen phosphate and glucose were of analytical grade.

#### 2.1.2. Standard Calibrator fAGEs-BSA Hydrothermal Synthesis and Fluorescence Characterization

For fAGEs-BSA, we used the protocol of Bhatwadekar and Ghole (2005) with minor modifications. Control samples consisted of: 1 mL of Na_2_HPO_4_ buffer (0.2 M; pH 7.4) alone, 1 mL of Na_2_HPO_4_ buffer (0.2 M; pH 7.4) with 0.5 M glucose and 1 mL of Na_2_HPO_4_ buffer (0.2 M; pH 7.4) with 50 mg/mL BSA, while experimental samples had: 1 mL of Na_2_HPO_4_ buffer (0.2 M; pH 7.4) with 0.5 M glucose and 50 mg/mL BSA. Experimental and control mixtures were prepared in triplicates and placed on a thermoblock in the dark for 96 h at 50 °C. Next, 4 μL of each sample was added in triplicate to black 96-well plates and diluted with 196 μL of Na_2_HPO_4_ buffer (0.2 M; pH 7.4) to final volume of 200 μL. Fluorescence characterization (Tecan Infinity m1000Pro microplate reader) was performed using an emission fluorescence spectrum in the range 390–600 nm and an excitation wavelength of 360 nm. Presence of fAGEs was detected around 440 nm in the emission fluorescence spectrum.

#### 2.1.3. fAGEs-BSA Calibration Curve

Synthesized fAGEs-BSA, and control BSA after the hydrothermal synthesis, were diluted in the concentration range of 0–100 µg/mL using Na_2_HPO_4_ buffer (0.2 M; pH 7.4) to the volume of 200 μL. Samples were loaded on black 96-well plates and fluorescence was recorded using an excitation wavelength of 360 nm and an emission wavelength of 440 nm. Based on the obtained relative fluorescence intensity (RFI) and the change in fAGEs-BSA concentration, we constructed a calibration curve, which was used for quantification of fAGEs in samples extracted from flies’ tissue.

#### 2.1.4. Influence of Monoamines, Psychostimulants and Antioxidant on fAGEs-BSA In Vitro Formation

We tested the influence of monoamines 3,4-dihydroxy-L-phenylalanine (L-DOPA), dopamine (DA), tyramine (TYRA), octopamine (OCT), tryptophan (TRY), psychostimulants cocaine (COC) and methamphetamine (METH) and antioxidant quercetin (QUE), on the fAGEs-BSA formation. Procedure for the effect of these compounds on the quantification of fAGEs-BSA was the same as described above for the experimental samples, but in the presence of 10 mM of tested substance. Percentage of fAGEs-BSA inhibition was calculated as the ratio of the fAGEs-BSA concentration generated in the presence of tested substance versus the fAGEs-BSA concentration generated in the standard calibrator.

### 2.2. In Vivo Experiments

#### 2.2.1. Chemicals and Reagents

3-iodo-L-tyrosine (3IY) (≥95%) and Triton X-100 were purchased from Sigma Aldrich. Trypsin-EDTA 0.025% solution was purchased from Lonza. All other chemicals used for PBS X1 preparation NaCl, KCl, Na_2_HPO_4_, KH_2_PO_4_ and HCl (36%) were of analytical grade. Other chemicals that we used are listed in the in vitro section.

#### 2.2.2. Fly Husbandry and Strains

We used 3 to 5 day-old male flies of the following genotypes: wild type (*wt*) flies in *CantonS* background (kind gift from C. Helfrich Forster, University of Würzburg, Germany), mutant flies lacking dopamine transporter (DAT) *fumin* (*fmn*) [36] and mutants lacking dopamine receptor type 1 (DR1) *dumb* [37]. Flies were raised in bottles containing standard cornmeal/agar medium at 25 °C and 70% humidity on a 12 h light/12 h dark cycle. Fly food recipe contained: 15 g of table sugar, 35 g of dry yeast, 12 g of Agar type I and 60 mL of 50% molasses and 970 mL of tap water. To prevent mold growth, we used 7.5 mL of p-hydroxybenzoic acid methyl ester (NIPAGINE, Roth, 99%) in 95% ethanol and 7.5 mL of propionic acid (Sigma Aldrich, 99%).

#### 2.2.3. Oral Administration of Substrates

Groups of 15 male *wt* flies were placed in plastic vials containing fly food with the addition of the following compounds and kept on it for 48 h in an incubator (25 °C; 70% humidity; 12 h light/12 h dark cycle): COC 0.5 mg/mL, METH 0.5 mg/mL, L-DOPA 1 mg/mL, 3IY 5 mg/mL, QUE 3.2 mM or combination of METH 0.5 mg/mL and QUE 3.2 mM, while male *fmn* mutant flies were fed with COC 0.5 mg/mL or METH 0.5 mg/mL.

#### 2.2.4. Sample Preparation for fAGEs Determination

Five male flies were placed in weighed and labelled empty Eppendorf tubes, in triplicate for each condition and genotype. Tubes were placed in an ice bath and whole body tissue samples were mechanically homogenized. We used two protein extraction buffers: Phosphate Buffered Saline (PBS) alone, for the extraction of extracellular proteins, or with addition of 0.1% Triton X-100 (PBT) for the extraction of total proteins. We used the ratio 5 mg tissue: 300 μL buffer. Homogenates were kept on ice for 30 min and centrifuged (30 min at 4 °C and 14.000 rpm). Supernatants were extracted to empty Eppendorf tubes and fAGEs were quantified. To test the influence of protein concentration on the fAGEs amount, we used PBT extraction buffer with 10 mM EDTA and 0.05 mg/mL of Trypsin to digest the samples for 24 h at 37 °C. Quantification of fAGEs was done using a Tecan Infinite 200 PRO microplate reader. 4 μL of body extract samples were loaded in 96 well plates, in triplicate, and diluted with 196 μL of Na_2_HPO_4_ (0.2 M; pH 7.4). The fluorescence emission was obtained using an excitation wavelength of 360 nm, and an emission wavelength of 440 nm. Concentration of fAGEs in the samples was calculated using a calibration curve.

### 2.3. Statistical Analysis

Statistical analysis was performed using GraphPad Prism (9.0.2) and results are expressed as mean ± standard error mean (SEM). Pearson’s correlation analysis was used to examine the significance of the relationship between fAGEs-BSA concentration (μg/mL) and RFI with λ_ex_360 nm and λ_em_440 nm. Linear regression analysis was used to calculate the R^2^ value from calibration curve results for interpolation/extrapolation of fAGEs concentrations in the samples. One-way ANOVA with the Bonferroni post hoc test was used for comparison of three or more unmatched groups. Two-way ANOVA with Bonferroni post hoc test was used for dependent variables at multiple levels of two categorical independent variables. Significant values in all figures: *: *p* < 0.05, **: *p* < 0.01, ***: *p* < 0.001.

## 3. Results

### 3.1. In Vitro fAGEs-BSA Hydrothermal Synthesis as Standard Calibrator for In Vivo fAGEs Quantification

**Figure 1:**
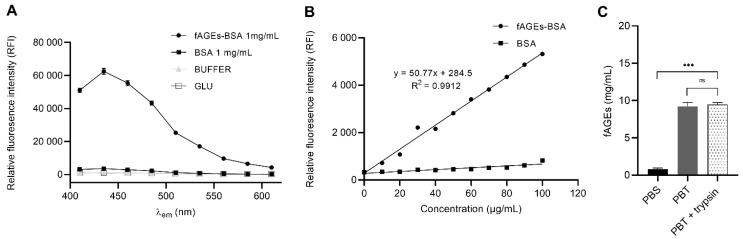
Quantification of fluorescent Advanced Glycation End products (fAGEs)in vivo from whole body samples based on in vitro hydrothermal synthesis of standard calibrator fAGEs-BSA (bovine serum albumin). (**A**) The fluorescence emission spectrum was recorded at 410–610 nm, using an excitation wavelength of 360 nm. 200 μL of samples: fAGEs-BSA, BSA, glucose and Na_2_HPO_4_ buffer, was measured in triplicate and shown as mean ± standard error mean (SEM). (**B**) Calibration curve for fAGEs-BSA and BSA alone as control in μg/mL concentration range with relative fluorescence intensity (RFI) recorded at λ_ex_360 nm; λ_em_440 nm (*r* = 0.9956; *n* = 11, in triplicate; Pearson’s correlation) and linear regression y = 50.77x + 284.5; R^2^ = 0.9912. (**C***)* in vivo quantification of fAGEs in the whole body homogenates of *Drosophila* males (*n* = 9) measured in triplicates, plotted as mean ± SEM for each of three conditions. Samples were prepared using: PBS buffer for the extraction of the extracellular proteins, PBT buffer for the extraction of the extracellular and cytosolic proteins, PBT + trypsin buffer for the proteolysis of the total extracellular and cytosolic proteins. Significant values: ***: *p* < 0.001 and non-significant (ns).

In order to produce a standard calibrator for fAGEs determination using fluorescence in *Drosophila* homogenate of the whole body, we prepared fAGEs-BSA by incubating BSA and the reducing sugar glucose (GLU) for 96 h at 50 °C. Measuring the fluorescence emission spectrum from 410–610 nm with λ_ex_360 nm, we successfully identified a fluorescence emission peak at around 440 nm for a product of a hydrothermal reaction between GLU and BSA (fAGEs-BSA 1 mg/mL), which was not present in the control samples of either BSA, buffer (Na_2_HPO_4_) or GLU alone (Figure 1A). To create a calibration curve, we recorded and plotted relative fluorescence intensity (RFI) for fAGEs-BSA and BSA in the concentration range from 0 to 100 μg/mL (Figure 1B). We obtained a positive correlation between fAGEs-BSA concentration and RFI at λ_ex_360 nm and λ_em_440 nm (*p* < 0.0001; *r* = 0.9956), while increasing concentration BSA in the control sample did not increase the RFI (Figure 1B). Correlation coefficient R^2^ for the fAGEs-BSA was 0.9912, which allowed us to precisely determine the concentration of fAGEs in the samples of unknown concentration.

To quantify fAGEs in *Drosophila* we used whole body homogenates of 3–5-day old wild type male flies of *CantonS* background. To test if total protein concentration affects fAGEs concentration, we used trypsin digestion, and showed that the fAGEs quantity is independent of protein digestion (Figure 1C). The total fAGEs amount consists of mostly cytosolic proteins based on results showing that contribution of extracellular proteins extracted using PBS buffer represents a small fraction of the total PBS extracted proteins (cytosolic and extracellular) (Figure 1C).

### 3.2. Effect of Monoamines and Psychostimulants on In Vitro fAGEs-BSA Formation

**Figure 2:**
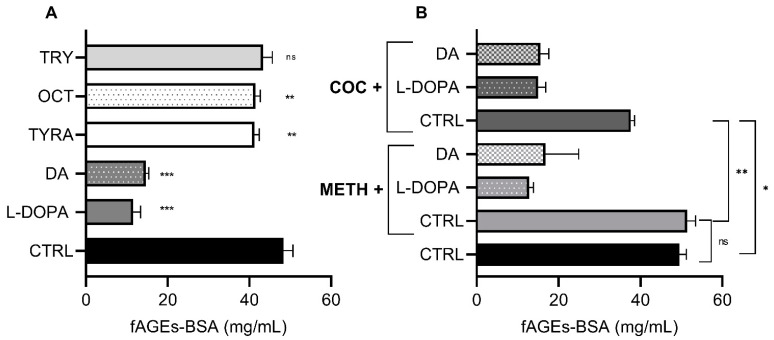
fAGEs-BSA formation is inhibited by 3,4-dihydroxy-L-phenylalanine (L-DOPA) and dopamine (DA) in vitro, regardless of the presence of cocaine (COC) or methamphetamine (METH). All samples were prepared in triplicates and all replicates were measured three times (*n* = 9). Data are presented as mean ± SEM. (**A**) CTRL—concentration of fAGEs-BSA after hydrothermal synthesis with 50 mg/mL BSA, 0.5 M glucose and Na_2_HPO_4_ buffer and with addition of: 10 mM of L-3,4-dihydroxyphenylalanine (L-DOPA), dopamine (DA), tyramine (TYRA), octopamine (OCT) or tryptophan (TRY). Significant values: **: *p* < 0.01, ***: *p* < 0.001 and non-significant (ns). (**B**) Concentration of fAGEs-BSA after hydrothermal synthesis with 50 mg/mL BSA, 0.5 M glucose and Na_2_HPO_4_ buffer (CTRL) and in the presence of 10 mM methamphetamine (METH CTRL) and 10 mM cocaine (COC CTRL). Other samples in addition contained 10 mM of L-3,4-dihydroxyphenylalanine (L-DOPA) or dopamine (DA). Significant values: *: *p* < 0.05, **: *p* < 0.01 and non-significant (ns).

To test if DA affects fAGEs formation in vitro, we introduced DA and other monoamines during the fAGEs-BSA synthesis (Figure 2A). L-DOPA and DA had the biggest influence on fAGEs-BSA, decreasing the formation by 76.13% and 69.78% respectively, relative to the buffer control (*p* < 0.0001). Tyramine (TYRA) and octopamine (OCT) had a less significant effect (decrease of 14.85% and 14.32%) (*p* < 0.01), while tryptophan (TRY) had a negligent effect.

In model organisms and humans, psychostimulants such as methamphetamine (METH) and cocaine (COC) increase monoaminergic signaling and lead to subsequent disruptions in monoaminergic metabolism [25,38]. To test if COC or METH have chemical properties that influence the amount of fAGEs-BSA formation in vitro, we measured the influence of COC or METH alone, or in conjunction with L-DOPA and DA. METH alone had no significant effect, while COC alone led to 22% inhibition of fAGEs-BSA formation (Figure 2B). Since L-DOPA and DA alone inhibited fAGEs-BSA formation (Figure 2A), we hypothesized that they will have a similar effect when applied together with COC or METH. Indeed, co-application of L-DOPA or DA significantly lowered fAGEs-BSA formation (Figure 2B). However, the amount of fAGEs-BSA was similar when L-DOPA and DA were applied alone or in conjunction with COC or METH (Figure 2A). This suggests that the amount of fAGEs-BSA reflects the action of L-DOPA or DA alone, and is not dependent on the presence of COC or METH.

### 3.3. Effect of DA Synthesis and Signaling on In Vivo fAGEs Formation

**Figure 3:**
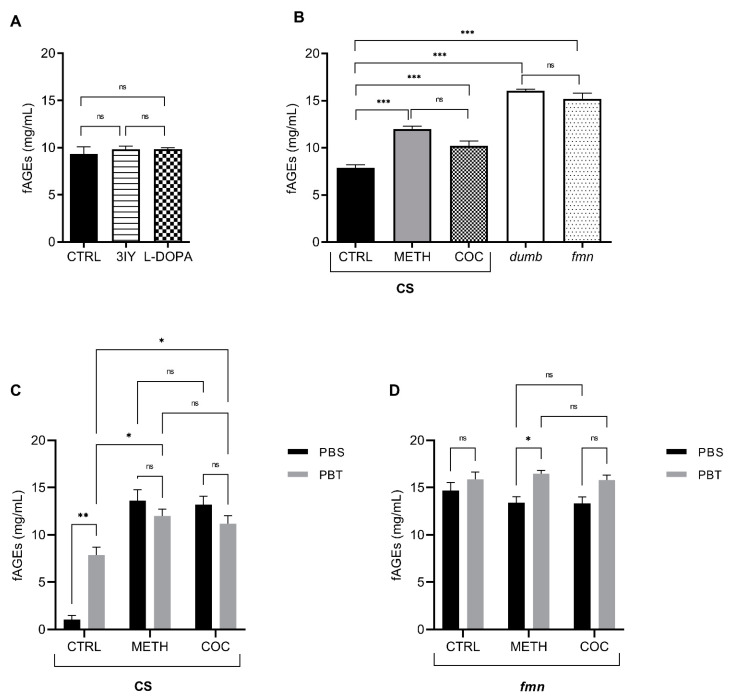
The amount of fAGEs formation depends on the type, duration and location of DA signaling manipulation. (**A**) fAGEs concentration in *wt* male whole body extracts prepared using PBT protocol: no treatment (CTRL), 48 h of feeding with 5 mg/mL 3-iodotyrosine (3IY) or 1 mg/mL L-3,4-dihydroxyphenylalanine (L-DOPA) (*n* = 5 flies for each condition, in triplicates; one-way ANOVA with Bonferroni post hoc tests, non-significant (ns)). (**B**) fAGEs concentration in male *wt* (CS), *dumb* and *fmn* whole body extracts using PBT protocol: CTRL *wt* flies without treatment, *wt* flies fed 0.5 mg/mL METH or COC for 48 h (*n* = 5 flies for each condition, in triplicates, one-way ANOVA with Bonferroni post hoc tests). Significant values: ***: *p* < 0.001 and non-significant (ns). (**C**) fAGEs concentration determined in whole body extracts using PBS and PBT protocols in CantonS *wt* male flies (CS): no treatment (CTRL) and fed for 48 h with 0.5 mg/mL METH or COC (*n* = 5 flies for each conditions, in triplicates, two-way ANOVA with Bonferroni post hoc tests). Significant values: *: *p* < 0.05, **: *p* < 0.01 and non-significant (ns). (**D**) fAGEs concentration determined in whole body extracts using PBS and PBT protocols in *fmn* mutant flies: no treatment (CTRL) and fed for 48 h with 0,5 mg/mL METH or COC (*n* = 5 flies for each conditions, in triplicates, two-way ANOVA with Bonferroni post hoc tests). Significant values: *: *p* < 0.05 and non-significant (ns).

Based on the reports that DA degradation in vivo leads to production of reactive oxidative species [39], we first tested how the change in the DA synthesis affects fAGEs quantity. We fed flies for 48 h with 5 mg/mL 3-iodotyrosine (3IY) to inhibit rate-limiting enzyme in DA synthesis, tyrosine hydroxylase, and we increased the DA amount by feeding flies 1 mg/mL L-DOPA, a dopaminergic precursor [40]. Neither of the two treatments influenced the fAGEs formation compared to the non-treated flies (Figure 3A).

Since the modulation of DA synthesis did not produce change in fAGEs formation, we tested if pharmacological or genetic manipulation of DA signaling influences fAGEs abundance. We fed male *wt* flies for 48 h with 0.5 mg/mL METH or 0.5 mg/mL COC. Both psychostimulants lead to increased fAGEs formation compared to unfed *wt* control (Figure 3B). To determine if fAGEs accumulation is due to the modification of total or extracellular proteins, we used PBS and PBT protein extraction protocols. In METH and COC fed flies, there is a significant increase in the fAGEs amount measured using PBS extraction protocol, compared to the control flies. This led to a non-significant change between fAGEs amounts derived using PBS and PBT protocols. These results show that the major effect of COC or METH on formation of fAGEs can be explained with the modification of extracellular proteins (Figure 3C).

Genetic disruption of DA transport and signaling was done using two genetic mutants: *dumb* is D1-type dopamine receptor mutant, which leads to decreased DA levels in the synaptic cleft [37], and *fumin* (*fmn*) is a dopamine transporter gene mutant, which results in increased DA levels in the synaptic cleft [36]. Both mutants had significantly higher amounts of fAGEs compared to *wt* flies or COC and METH fed flies (Figure 3B).

To determine the contribution of extracellular to total proteins modification, we exposed *fmn* flies to 0,5 mg/mL of COC and METH and measured fAGEs after PBS and PBT protein extraction. Control *fmn* flies have increased fAGEs as a consequence of extracellular protein glycation (Figure 3D). METH and COC feeding led to no additional increase in fAGEs concentration, emphasizing the extracellular effect measured with PBS protocol.

### 3.4. Influence of Quercetin on In Vivo fAGEs Formation

**Figure 4:**
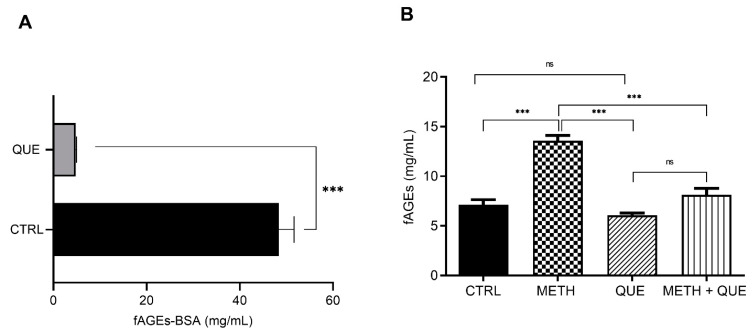
METH induced fAGEs-BSA formation in vitro is inhibited by the antioxidant QUE. (**A**) fAGEs-BSA hydrothermal synthesis with 50 mg/mL BSA, 0.5 M glucose and Na_2_HPO_4_ buffer alone (CTRL) or with 10 mM quercetin (QUE). All samples were prepared in triplicates and all replicas were measured three times (*n* = 9). Data are presented as mean ± SEM. Significant values: ***: *p* < 0.001. (**B**) fAGEs concertation in *wt* male whole body extracts prepared using PBT protocol: (CTRL) mock treated, (METH) fed 0.5 mg/mL METH alone or 3.2 mM QUE alone (QUE) or in combination (METH + QUE) for 48 h (*n* = 5 flies for each condition, in triplicates). Significant values: ***: *p* < 0.001 and non-significant (ns).

We tested the influence of a known flavonoid and antioxidant, quercetin (QUE), on fAGEs formation in vivo and in vitro. QUE significantly inhibited fAGEs-BSA formation in vitro (90.05% decrease relative to control) (Figure 4A). To explore if QUE has a similar effect in vivo in conditions that are known to increase oxidative stress, we fed *wt* flies METH or QUE alone, or in combination. We confirmed that METH feeding increases fAGEs concentration in vivo. However, QUE feeding alone had no effect on fAGEs, which is in contrast to in vitro results (Figure 4A,B). Combined feeding of METH and QUE led to a significant decrease in fAGEs relative to METH alone, suggesting that QUE antagonizes METH-induced fAGEs formation.

## 4. Discussion

To investigate if fAGEs are dependent on, and vary with DA synthesis and signaling, we performed a series of in vitro and in vivo experiments. in vitro fAGEs-BSA formation was inhibited in the presence of DA, in contrast to the lack of effect in *Drosophila*. Increase in DA signaling in *Drosophila* using psychostimulant METH or COC significantly increased fAGEs quantity in vivo, although they did not affect fAGEs-BSA formation in vitro. In *fmn* and *dumb Drosophila* mutants, the fAGEs amount was higher than when flies were fed COC or METH. Increased fAGEs in *fmn* flies, and *wt* and *fmn* flies fed COC and METH, is due to the glycation of extracellular proteins. Finally, the METH induced increase in fAGEs formation was reduced by application of the antioxidant QUE.

We studied dopaminergic effects on fAGEs formation in vivo and in vitro because of DA’s dual nature. First, DA is a highly reactive catecholamine that can be easily oxidized under biological conditions, either enzymatically or non-enzymatically [39]. DA can be metabolized intracellularly by monoamine oxidase-B, producing 3,4-dihydroxyphenylacetic acid (DOPAC) and hydrogen peroxide, which can react with transition metal ions to produce highly toxic hydroxyl radicals through the Fenton reaction. DA can also undergo autoxidation generating the superoxide anion, which in turn reacts with transition metal ions via the Haber–Weiss/Fenton reaction, generating hydroxyl radicals [39]. Second, in vitro DA can act as a strong antioxidant based on two hydroxyl groups in the position 1,2 on the phenolic ring with strong hydrogen-donating activity characteristic for ROS scavengers [41]. In many brain diseases such as addiction, neurodegeneration and neuropsychiatric disorders, changes in brain function involve disruption of the dopaminergic system, and consequentially, the redox balance.

fAGEs are a subgroup of AGEs molecules with auto-fluorescent properties [10,11,42], which can be used as an indicator of impaired oxidative defense. In order to produce a standard calibrator for quantifying fAGEs amount from the *Drosophila’s* tissue, we have synthesized fAGEs-BSA. Using a modified protocol [34,35] for the in vitro synthesis of fAGEs-BSA, we have confirmed the product using fluorescence spectroscopy with λ_ex_360 nm and λ_em_440 nm, and showed concentration-dependent linear increase in the fluorescence of fAGEs-BSA.

*Drosophila* was previously used for studying fAGEs in the context of aging when fAGEs was measured in the whole body extracts, but without a standard calibrator [1,2]. Using fAGEs-BSA as a standard calibrator, we have precisely quantified the fAGEs amount in tissue extracts of *wt* flies, and using PBS versus PBS extraction protocols, have shown that they are abundant in cellular extracts, indicating modification of cellular proteins. Furthermore, total fAGEs concentration does not change when PBT protocol includes trypsin; thus, the total fAGE amount is independent of protein cleavage.

One of the important outcomes from our study is the difference between DA effects on fAGEs-BSA formation in vitro, versus fAGEs accumulation in vivo. We show that dopaminergic precursor L-DOPA or DA inhibits fAGEs-BSA formation, which is in agreement with the antioxidant property of DA in vitro [41]. However, increased DA synthesis in vivo after L-DOPA feeding likely leads to the increased vesicular pool in the presynaptic neuron and potentially increased release of DA upon stimulation [43,44,45]. Thus, our expectation was that we will observe an increase in fAGEs amount in vivo. A potential explanation why we did not observe it is homeostatic regulation of dopamine signaling. Released synaptic DA is cleared either through transport back to the presynaptic neuron where it is packaged into vesicles to prevent auto-oxidation of DA [32], or is oxidized to dopamine quinone [39], as both mechanisms are aimed at terminating the stimulation of the postsynaptic neuron [46]. Thus, while L-DOPA and 3IY change the amount of DA in the cell [40], they engage mechanisms that control the dopaminergic system during normal functioning. Mechanisms that regulate dopaminergic homeostasis in vivo consequently influence dopaminergic pro- and antioxidant property, unlike in vitro environment, where such regulation does not exist.

An alternative, or potentially complementary explanation, of the in vitro results has to do with BSA molecular structure. In the tertiary structure, BSA has 17 interchain disulfide bonds formed by 34 oxidized cysteines and 1 free sulfhydryl group in one reduced cysteine [47]. The heating procedure denatures the BSA tertiary structure and influences disulfide bond reactivity [48]. DA and oxidized dopamine quinone could be covalently added on free cysteine residues [49,50]. We have observed a peak of the fluorescence emission spectrum at 485 nm (data not shown) which was present only in in vitro DA and L-DOPA samples, but not in other tested monoamines. This suggested to us a direct binding of free DA and L-DOPA to cysteine in BSA, which interferes with glucose binding on the free amino groups and results in the inhibition of fAGEs-BSA formation.

COC and METH have psychoactive properties in large part due to their effect on the dopaminergic system. COC prevents DA uptake into the presynaptic neuron by blocking the activity of the monoaminergic transporters. METH enters the presynaptic neuron and monoaminergic vesicles, causing their release into the cytosol and then into the synaptic cleft [38,51]. In mammals, COC can be hydrolyzed in the synaptic cleft to benzoate and ecgonine, and both can be covalently added to the protein structure [52]; however, COC does not cause intracellular disruption of the DA metabolism to the same extent as METH. These differences agree with our results that show a higher increase in the amount of fAGEs formation following METH feeding, compared to COC feeding (Figure 3B). We speculate that the increased amount of cytosolic DA after METH feeding induces oxidation and leads to increased fAGEs.

Our results agree with the increased dopaminergic signaling after COC and METH feeding in *wt* flies. We predicted that increased extracellular DA will undergo oxidative metabolism and increase extracellular protein glycation. We confirmed that by showing that there is a significant increase in the fAGEs quantity using the PBS extraction protocol, indicating that psychostimulants mostly lead to modification of extracellular proteins. Similarly, *fmn* mutant flies that have increased dopaminergic signaling also show that fAGEs accumulation in total protein extracts is derived from the extracellular fraction. COC or METH feeding of *fmn* flies did not lead to further change in the extracellular fAGEs amount. This finding is in line with the measurement of extracellular DA in *fmn* mutant after COC exposure, where it was shown that COC does not lead to further increase in the extracellular DA [53]. In METH fed *fmn* flies, we have observed a small but significant increase of the fAGEs amount in total protein extracts compared to extracellular, which agrees with the METH effect on monoaminergic vesicles and subsequent increase in the cellular DA.

METH had no significant effect on fAGEs-BSA in vitro, which is expected because of the absence of the enzymatic environment otherwise present in vivo. A small effect of COC on decreased fAGEs-BSA formation in vitro can only be explained with the addition of COC hydrolyzation products to the BSA molecule that ultimately lowers fAGEs-BSA [52].

DA undergoes metabolic and oxidative transformations through enzymatic and non-enzymatic mechanisms. Oxidative damage accumulates over time, and the temporal effect on fAGEs formation is evident as a difference between COC and METH feeding, versus the amount of fAGEs in genetic mutants. Disruption of dopaminergic signaling in *fmn* and *dumb* flies is present throughout their lifetime, while flies are fed COC and METH only for 48 h. The lifelong disruption of dopaminergic signaling leads to disruption in the intracellular mechanism of DA storage and release that potentiates oxidative reactions [54]. *dumb* flies have a lower DA release that can lead to the oxidative stress [55], which is supported by our finding of increased fAGEs amount in these flies compared to *wt* flies. We propose that changes in fAGEs formation after COC and METH feeding, and in genetic mutants with the disruption in the dopaminergic signaling, are due to oxidative changes induced by dopaminergic metabolism.

This argument is further supported by the in vitro and in vivo experiments with QUE. Plant flavonoids inhibit fAGEs formation [56], as has been shown for QUE [17]. In our study, QUE in vitro significantly lowered fAGEs-BSA, supporting its antioxidative effect. QUE feeding alone, however, did not change fAGEs, and that could be a consequence of the dose in combination with the homeostatic regulation of redox balance in vivo. However, QUE significantly lowered METH-induced fAGEs increase supporting the antioxidant nature of QUE, likely by modulating oxidative effects of increased dopaminergic signaling.

Using in vivo and in vitro manipulations, we have correlated changes between fAGEs abundance and DA oxidative metabolism. In *wt* flies, extracellular fAGEs contributes little to the total protein extracted from *Drosophila’s* tissue, which agrees with the fact that metabolism and the consequent oxidation happens in the cell and that dopaminergic signaling is tightly regulated through recycling mechanisms. In flies where we increased dopaminergic signaling, through psychostimulant feeding or in the genetic mutants, fAGEs formation was predominant in the extracellular fraction. This suggests that oxidation of excessive amounts of DA present in the synaptic cleft leads to modification of extracellular proteins and formation of fAGEs. The potential methodological improvements that follow from our work are: first, our in vitro method can be used for the study of other model proteins and reducing sugars in order to describe the complexity of physiological conditions where glycation affects protein function; and second, fAGEs auto-fluorescence properties can be optimized and used for non-invasive surface detection in flies.

## Data Availability

The data that support the findings of this study are available from the corresponding author upon reasonable request.
